# Classification of Interdental Space for Different Quadrants on the Basis of Standardization through Threshold Data and Its Comparison with BMI and Socioeconomic Status

**DOI:** 10.5005/jp-journals-10005-1179

**Published:** 2013-04-26

**Authors:** Tapan Singh, Ronauk Singh, Jatinder Pal Singh

**Affiliations:** Postgraduate, Department of Pedodontics, Saraswati Dental College, Lucknow, Uttar Pradesh, India; Captain, Department of Prosthodontics, Army, West Bengal, India; Major, Department of Prosthodontics, Army, Maharashtra, India

**Keywords:** Primary dentition, Socioeconomic

## Abstract

**Background:** A better knowledge about the Interdental space is important since it provides insights on the prevalence of malocclusion. To date, there is conflicting evidence on the impact of body mass index (BMI) and Socioeconomic status (SES) on interdental space. A recent review concluded that a greater understanding is required of the interdental space. Therefore, there is a need for a more comprehensive and rigorous assessments of the interdental space and impacts of BMI and SES.

**Aim:** BMI and SES can be associated with the interdental spacing in deciduous dentition.

**Design:** The present cross-sectional study was carried out on 448 children of age group of 3 to 5 years out of which 392 were meeting our criteria. Research assessment questionnaire on demographic data was completed by the parents. Study model cast of 392 children free from malocclusion were analyzed.

**Results:** A statistically significant association between interdental spacing and BMI category was observed. Comparison of BMI with above threshold interdental space revealed that after an optimum weight there is no effect on interdental space.

A significant association between SES and interdental spacing was observed for all the four locations (p < 0.01).

**Conclusion:** Evolved normative value can be taken as a standard and the occlusion and interdental spaces are not two completely separate entities and they are interdependent.

**How to cite this article:** Singh T, Singh R, Singh JP. Classification of Interdental Space for Different Quadrants on the Basis of Standardization through Threshold Data and Its Comparison with BMI and Socioeconomic Status. Int J Clin Pediatr Dent 2013;6(1):16-21.

## INTRODUCTION

Primary dentition is believed to provide basis for studying occlusion and for predicting the occlusion of the permanent dentition. In reality, dental occlusion is a much more complex relationship but in its simplex form occlusion and can be defined as ‘the static relationship between the incising or masticating surfaces of the maxillary or mandibular teeth or tooth analogs’.^1^ Bogue^2^ quoted that if malocclusion was found in the primary dentition, it was to be expected that the same irregularities would occur in the corresponding permanent dentition–only to a more pronounced degree.^3-5^ Spacing was first described by Dellabarre in the year 1819 in deciduous dentition between the ages 4 and 6 years.^6^ Spacing in the deciduous dentition has been called physiological spaces by Korkhans and Newmann and developmental spacing by Graber.^7^

Absence of spaces in the primary dentition is an expression of disproportion between jaws/tooth size. The understanding of the anteroposterior changes that occur in the occlusion between the deciduous and permanent dentition is crucial for the clinician, involved in early orthodontic treatment. According to Reddy^8^ it was found that there is a significant relationship between body mass index (BMI) and socioeconomic status (SES). An another study carried out by Thomaz and Valenca^9^ found out association between weight for age (W/A) and increase chance of dental crowding in children aged 3 to 5 years; thus it would also be logical to assume that there is relationship between BMI and physiological spacing and also between SES and physiological spacing. Keeping the above discussion in view the present study was envisaged.

## METHODOLOGY

**Graph 1:**
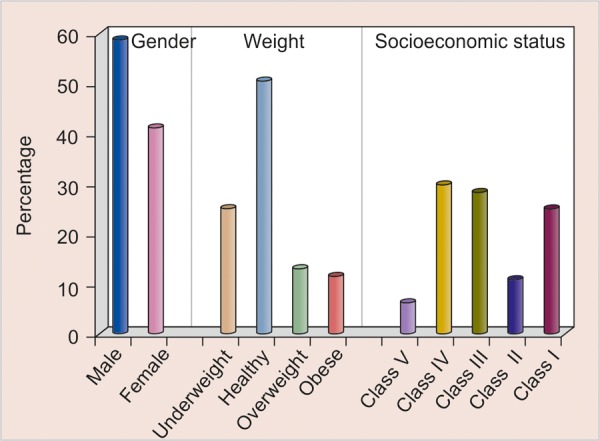
Dermographic distribution of study subjects

The present cross-sectional study was carried out on 448 children of age group of 3 to 5 years out of which 392 were meeting our criteria. The information was recorded on a self-prepared questionnaire. The age of the child was obtained from school records. Social class was determined as per Prasad's classification (1970) with price index correction of 2002; BMI was calculated as per CDC criteria. Dermographic distribution of the study subjects are illustrated in Table 1 and Graph 1. The children were selected following an oral examination performed under natural day light using mouth mirror with good reflecting surface and stainless steel explorer. A full depth alginate impression of maxillary and mandibular arches of each child was made using stainless steel impression trays. Impressions were washed under running tap water and were disinfected using glutaraldehyde. Study model cast were made. All necessary aseptic measures and barrier techniques were followed throughout the study. Interdental spaces were measured on the study model cast using vernier caliper with 0.01 mm accuracy. At first, one of the beak was seated in the interdental space, if space remained between the teeth, another gouge (beak) was added to first one until the interdental space became completely filled with the beak without any pressure on teeth. Finally, overjet and overbite were measured in centric occlusion relationship.

**Table 1:** **Table 1:** Dermographic distribution of study subjects

*S.no.*	*Variable*	*Statistic*
1.	Mean age ± SD (range)	4.17 ± 0.90 (3-5)
2.	Male: Female	230 (58.7%):162 (41.3%)
3.	BMI category (CDC classification) Underweight Healthy Overweight Obese	98 (25%) 198 (50.5%) 51 (13.0%) 45 (11.5%)
4.	Socioeconomic status (BG Prasad's classification) Class V Class IV Class III Class II Class I	24 (6.1%) 117 (29.8%) 111 (28.3%) 42 (10.7%) 98 (25.0%)

### Evolution of Threshold Values

As the interdental spaces were evaluated in four quadrants, i.e. anterior mandibular, posterior mandibular, anterior maxillary and posterior maxillary. It was difficult to categorize the interdental spaces in an individual as normal, below normal or above normal on the basis of a single parameter. Moreover, the differences in values of interdental spaces in different quadrants were extreme. In the absence of a threshold range, in present study, this range was developed from the data of normal weight children. Classification of threshold values of interdental space has been shown in Table 2.

After categorizing an individual for different quadrants, the interdental space was classified as follows:

### Criteria

Threshold region = values in all the four quadrants falling in threshold range.

**Table 2:** **Table 2:** Classification of interdental space for different quadrants on the basis of standardization through threshold data

*S. no.*	*Quadrant*	*Interdental space values*	
		*Threshold region (≤95% CI of normal weight children)*	*Above threshold (>95% CI of normal weight children)*
1.	Maxillary anterior	<0.72-1.07	>1.07
2.	Mandibular anterior	<0.96-1.41	>1.41
3.	Maxillary posterior	<0.015-0.089	>0.089
4.	Mandibular posterior	<0.03-0.14	>0.14

Above threshold = values in one or more quadrant above threshold range.

## RESULT

The present study was carried out with an aim to evaluate the relationship between BMI and interdental spacing in upper and lower arches and relation between SES and

For this purpose, a total of 392 children aged between 3 and 5 years (mean age, 4.17 ± 0.90 years) were enrolled in the study. The demographic distribution of study subjects has been shown in Table 1.

## ANALYSIS ACCORDING TO NORMATIVE RANGE EVOLVED DURING THE COURSE OF STUDY

For all the locations except maxillary posterior arch, the prevalence of interdental space in threshold region was maximum in underweight category. For maxillary posterior arch the prevalence of interdental space in threshold region was maximum in normal weight category. Overall, the prevalence of interdental space above threshold was 42.7%.

The prevalence was minimum in underweight group (27.6%) and maximum in obese group. Statistically, there was a significant difference in prevalence of interdental space above threshold among different weight groups (p = 0.004).

### Relation between SES and Interdental Spacing in Upper and Lower Arches

No association of gender with interdental space was observed (p = 0.874). Prevalence of interdental space was found to be significantly associated with different molar relationships. It was observed that as compared to distal step and flush terminal molar relationships, the prevalence of above threshold range of interdental space was significantly higher in mesial step molar relationship (p < 0.001). In canine relationships, the prevalence of above threshold interdental space was significantly lower in class II canine relationship as compared to other two classes (p = 0.045). In different facial forms, the prevalence of interdental space was found to be significant statistically (p = 0.039) in brachiocephalic and mesocephalic forms as compared to dolichocephalic form. An association between SES and prevalence of above normative interdental space was observed. The prevalence was minimum in class III income group and maximum in class II group. The prevalence of above threshold interdental space was found to be significantly higher in overbite (>3 mm) and overjet (>3 mm) groups.

## DISCUSSION

**Graph 2:**
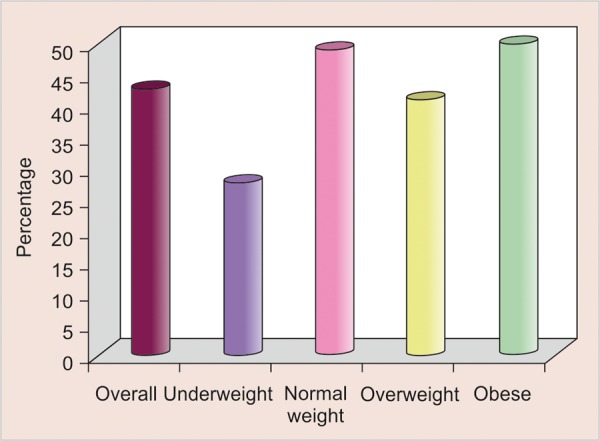
Relationship between BMI and interdental spacing

Analysis of the relationship between BMI and interdental spacing showed a statistically significant association in our study. In order to compare interdental space as a single entity with BMI, an evaluation of threshold range was done in the values of interdental space in all the four quadrants. This range was formed by calculating 95% confidence interval (CI) in normal weight children evolution of threshold range was done because the values of interdental space in all the four quadrants were extreme and interdental spaces cannot be classified on the basis of single parameter, i.e. BMI alone or gender or SES. So a criterion was formulated to classify interdental spaces which was: (1) Threshold level and (2) above threshold level. Relationship between interdental space (as per criteria evolved during the course of study) and BMI was evaluated. Data was organized in Tables 3A and B and illustrated in Graph 2. The data confirmed us that there is a statistical significant difference in the prevalence of above threshold interdental space between the underweight and normal weight children, with normal weight children showing greater prevalence. Similar readings were also observed in the category of overweight and obese when they were compared with underweight children. Hence, it can be concluded from the data that after optimum weight obtained there is no change in the prevalence of interdental spaces, which means that after normal weight category there is no statistical difference among all the three categories that are normal weight, overweight and obese. BMI can be used as a measurement scale for assessment of malnutrition in children.^10^ Hence, it can be hypothesized that malnutrition (underweight category) may be associated with decrease in interdental spacing. It was found that there was a significant adverse affect of malnutrition on the growth and development of facial bones and on the development of skeletal muscles.^11-13^ Altered bone growth in the craniofacial complex caused by poor nutrition could be reflected in reduced interdental spacing. There is some evidence in animal models that support the hypothesis of association between malnutrition and malocclusion using pigs^14,15^ and rats.^16^ This proves our hypothesis that BMI plays an important role in the prevalence of interdental spacing, which also support the study done by Thomaz.^9^ It indicates that malnutrition changes the growth pattern of the bones of the skeleton, including those of the face and oral cavity. The mechanisms that might explain this relationship have yet to be fully clarified. One line of reasoning is based on the restricted growth and development of bones in general (and of the bones of the face in particular) in the presence of malnutrition accompanied by stunting. These studies suggest that stunting could be reflected in, for example, the height of the mandible,^11^ the height of the lower face and width of dental arches.^17-20^ It is thus reasonable to hypothesize that low BMI can be associated with the restricted growth/development of the bones of the face and can change the amount of interdental spacing in deciduous dentition, rendering the association observed in this study more biologically plausible. Reasoning is also in the line of the functional matrix theory,^21,22^ according to which the face grows in response to functional needs throughout an individual's life. The shape of the dental arch would thus be strongly influenced by oral functions and response by the muscular pressure exerted on these tissues.

**Table 3A:** **Table 3A:** Quadrant-wise distribution of subject according to category of interdental space (as per criteria evolved during the course of study)

*Quadrant*	*Total (n = 392)*			*Underweight (n = 98)*			*Normal (n = 198)*			*Overweight (n = 51)*			*Obese (n = 45)*	
	*Threshold region*	*Above threshold*		*Threshold region*	*Above threshold*		*Threshold region*	*Above threshold*		*Threshold region*	*Above threshold*		*Threshold region*	*Above threshold*
Max ant	275 (70.2%)	117 (29.8%)		80 (81.6%)	18 (18.4%)		131 (66.2%)	67 (33.8%)		34 (66.7%)	17 (33.3%)		30 (66.7%)	15 (33.3%)
**X**^2^ = 8.232 (df = 3); p = 0.041														
Mand ant	284 (72.4%)	108 (27.6%)		80 (81.6%)	18 (18.4%)		130 (65.7%)	68 (34.3%)		40 (78.4%)	11 (21.6%)		34 (75.6%)	11 (24.4%)
**X**^2^ = 9.849 (df = 3); p = 0.020														
Max post	360 (91.8%)	32 (8.2%)		90 (91.8%)	8 (8.2%)		188 (94.9%)	10 (5.1%)		44 (86.3%)	7 (13.7%)		38 (84.4%)	7 (15.6%)
***_X_***^2^=7.944 (df = 3); p = 0.047														
Mand post	323 (95.2%)	5 (4.8%)		96 (98.0%)	2 (2.0%)		184 (92.9%)	14 (7.1%)		49 (96.1%)	2 (3.9%)		44 (97.8%)	1 (2.2%)
**X**^2^=4.563 (df = 3); p = 0.207														
Overall	224 (57.3%)	167 (42.7%)		71 (72.4%)	27 (27.6%)		101 (51.0%)	97 (49.0%)		30 (58.8%)	21 (41.2%)		22 (50.0%)	22 (50.0%)
**X**^2^ = 13.399 (df = 3); p = 0.004														

**Table 3B:** **Table 3B:** Between group comparison

*Underweight vs normal weight*		*Underweight vs overweight*		*Underweight vs obese*		*Normal weight vs overweight*		*Normal weight vs obese*		*Overweight vs obese*
**x**^2^ = 12.378;		**x**^2^ = 2.852;		**x**^2^ = 6.771;		**x**^2^ = 0.993;		**x**^2^ = 0.015;		**x**^2^ = 0.742;
p < 0.001		p = 0.091		p = 0.009		p = 0.319		p = 0.904		p = 0.389

SES is an important determinant of health and nutritional status. An attempt was made to determine the influence of SES on interdental spacing in our study.

Public schools are worst off socioeconomically, making t impossible to provide a suitable contrast for this variable, which is considered important in predicting oral health.^23,24^ So to overcome this flaw in the study, the sample collected were from both Public and Private schools. There have been several attempts to find out the social class of an individual. n Indian studies, Kuppuswamy (1962)^25^ scale is widely used to measure SES of an individual in urban community based on education, occupation and per capita income. In rural area Parekh^26^ (1964) classification based on nine characteristics has been used, but since our sample contained both urban and rural population we used Prasad's classification^27-29^ (1961) based on per capita income.

In the present study, a significant association between SES and interdental spacing was observed for all the four locations (p < 0.01).

**Graph 3:**
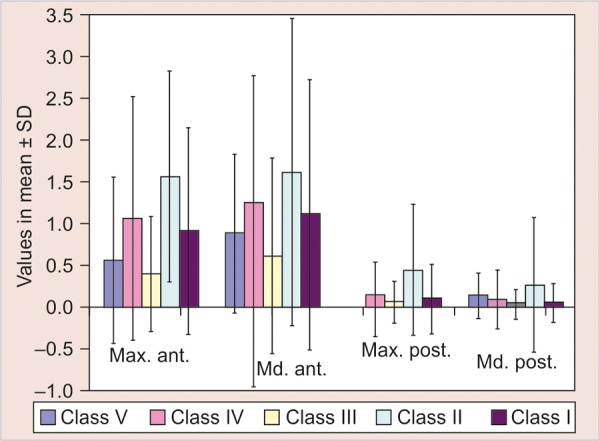
Relation between SES and interdental spacing

To find out specific trend in relationship between SES and interdental spacing a student t-test was used to find out the significance between the different classes in the SES classification. Graph 3 showed us that there is no specific trend. On observing Table 4 the prevalence of above threshold interdental space in class III income group was minimum and maximum in class II group. This establishes that SES cannot produce a direct relationship on interdental spacing and it can only be related through growth, which in turn is influenced by diet and nutrition. The prevalence of above threshold range of interdental space was significantly higher in mesial step molar relationship (p < 0.001) as compared to distal step and flush terminal molar relationships in both maxillary and mandibular arches.^30^ In the present study, the prevalence of different canine relationships with above threshold interdental space was significantly lower in class II canine relationship as compared to other two classes (p = 0.045) (Table 5). The prevalence of above threshold interdental space was found to be significantly higher in sample having overbite (>3 mm) and overjet (>3 mm). Proportion of total subjects with overbite and overjet >3 mm not falling into safe zone of =8 (2.05%) and 34 (8.7%) respectively, this means that in future children having interdental spacing in the range of above threshold will have less probability of malocclusion which was supported by a study done by Shivakumar.^31^ Therefore, it seems that occlusion and interdental spaces are not two completely separate entities and they are interdependent. A longitudinal research should be done to evaluate relationship between occlusion and interdental space from the time of primary teeth eruption till it reaches mixed dentition period. So, the confounding factors can diminish and the results will become more reliable and justifiable.

**Table 4:** **Table 4:** Relation between SES and interdental spacing

*SES*		*N*		*Max anterior*			*Mand anterior*			*Max posterior*			*Mand posterior*	
				*Mean*	*SD*		*Mean*	*SD*		*Mean*	*SD*		*Mean*	*SD*
Class V		24		0.50	0.91		0.79	0.87		0.00	0.00		0.11	0.25
Class IV		117		0.96	1.34		1.13	1. 71		0.12	0.37		0.07	0.32
Class III		111		0.35	0.63		0.55	1.07		0.04	0.22		0.02	0.16
Class II		42		1.42	1.16		1.47	1.69		0.38	0.73		0.23	0.74
Class I		98		0.82	1.14		1.00	1.48		0.08	0.38		0.03	0.21
F (ANOVA)				9.458			4.009			6.940			3.588	
p				<0.001			0.003			<0.001			0.007	

**Table 5:** **Table 5:** Association of different demographic and clinical variables with interdental space

*S.no.*	*Variable*	*Total no.*	*No. with interdental space above threshold*	*%*	*Significance or association*	
					***X**^2^*	*p*
1.	Gender					
	Male	230	99	43.0	0.025	0.874
	Female	161	68	42.2		
2.	Molar relationship					
	Distal step	35	8	22.9	28.166	<0.001
	Flush terminal	100	25	25.0		
	Mesial step	256	134	52.3		
3.	Canine relationship					
	Class I	327	146	44.6	6.193	0.045
	Class II	35	8	22.9		
	Class III	29	13	44.8		
4.	Facial form					
	Brachiocephalic	95	49	51.6	6.463	0.039
	Dolichocephalic	80	26	32.5		
	Mesocephalic	216	92	42.6		
5.	Socioeconomic status					
	Class V	24	14	58.3	48.733	<0.001
	Class VI	117	58	49.6		
	Class III	110	23	20.9		
	Class II	42	34	81.0		
	Class I	98	42	42.9		
6.	Overbite >3 mm	29*	21	72.4	11.294	0.001
7.	Overjet >3 mm	76**	42	55.3	6.074	0.014

*Proportion of total subjects with overbite >3 mm not falling into safe zone = 8 (2.05%); **Proportion of total subject with overjet >3 mm not faling into safe zone = 34 (8.7%)

## CONCLUSION

1. A statistically significant association between interdental spacing and BMI category was observed. Comparison of BMI with above threshold interdental space revealed that after an optimum weight there is no effect on interdental space.
2. A significant association between SES and interdental spacing was observed for all the four locations (p < 0.01)— on comparision of interdental spacing at different location between different SES groups revealed that there is no specific sequence in relationship between them.

## WHY THIS PAPER IS IMPORTANT TO PEDIATRIC DENTISTS?

1. It was earlier difficult to categorize the interdental spaces in an individual as normal, below normal or above normal on the basis of a single parameter. In the present study, this range categorization was developed from the data of normal weight children.
2. This study shows that occlusion and interdental spaces are not two completely separate entities and they are interdependent.
3. Study concludes from the data that after normal weight category there is no statistical difference among all the three categories that are normal weight, overweight and obese.
4. Study can predict that the children having interdental spacing in the range of above threshold will have less probability of malocclusion in future.
